# Effect of Ammonia on *Escherichia coli* Growth and Aerobic Respiration: The Role of Cytochrome *bd*-II

**DOI:** 10.3390/antiox15070859

**Published:** 2026-07-09

**Authors:** Francesca Giordano, Martina Roberta Nastasi, Vitaliy B. Borisov, Elena Forte

**Affiliations:** 1 Department of Biochemical Sciences, Sapienza University of Rome, I-00185 Rome, Italy; f.giordano@uniroma1.it (F.G.); martina_nastasi@live.it (M.R.N.); 2 Belozersky Institute of Physico-Chemical Biology, Lomonosov Moscow State University, Leninskie Gory, 119991 Moscow, Russia; 3 Faculty of Bioengineering and Bioinformatics, Lomonosov Moscow State University, Leninskie Gory, 119991 Moscow, Russia

**Keywords:** bacteria, redox enzymes, respiratory oxidases, ammonia, environmental stressor

## Abstract

Bacterial terminal oxidases are essential for growth and may provide protection against environmental stressors. Bacteria often have to cope with ammonia, which, although an essential nutrient, is toxic at high concentrations. Here, we studied the influence of ammonia on the cell growth of three different *Escherichia coli* respiratory mutants, each possessing a single terminal oxidase: cytochrome *bd*-I, cytochrome *bd*-II, or cytochrome *bo*_3_. We also investigated the effect of ammonia on O_2_ consumption in cytochrome *bd*-II-only cells and membranes, as well as in the isolated *bd*-II enzyme. Using microcalorimetry, spectrophotometry and high-resolution respirometry, the following new results were obtained: (i) At pH 8.3, the addition of ammonia to both *bd*-I-only and *bd*-II-only cell cultures has virtually no effect on growth. In contrast, the growth of *bo*_3_-only cells is significantly impaired. (ii) The addition of ammonia to *bd*-II-only intact cells at pH 8.3 not only fails to inhibit their respiration but also accelerates O_2_ consumption. The same is observed with *bd*-II-only isolated membranes and detergent-solubilized *bd*-II enzyme. The maximum increase in cytochrome *bd*-II O_2_ consumption rate is approximately 150%. Physiological aspects of the findings are discussed, and molecular mechanisms for ammonia-induced acceleration of O_2_ consumption by cytochrome *bd*-II are suggested.

## 1. Introduction

The respiratory chain of *Escherichia coli* is branched and flexible [1,2,3]. Depending on growth conditions, *E. coli* can express up to three terminal oxidases—cytochrome *bd*-I, cytochrome *bd*-II, and cytochrome *bo*_3_. Cytochrome *bo*_3_ predominates under conditions of high aeration. Cytochrome *bd*-I is upregulated under low oxygen tension. Cytochrome *bd*-II is induced during the transition from microaerobiosis to anaerobiosis, as well as under carbon and phosphate starvation [4]. Cytochrome *bo*_3_ is a member of the heme-copper oxidase superfamily, which also includes cytochromes *aa*_3_, *ba*_3_, *caa*_3_, and *cbb*_3_ [5,6,7]. Cytochrome *bd*-I and cytochrome *bd*-II belong to the *bd*-type oxidase superfamily, which is different from the heme-copper oxidase superfamily [8,9].

The three *E. coli* oxidases catalyze the four-electron oxidation of O_2_ by ubiquinol-8 and/or menaquinol-8 [4]. Part of the free energy released in this exergonic reaction is conserved in the form of a proton motive force [10,11]. The three-dimensional structures of the enzymes were resolved [12,13,14,15,16,17,18,19,20]. Cytochrome *bo*_3_ contains a ubiquinol binding site, heme *b*, and a binuclear site for O_2_ reduction composed of heme *o*_3_ and Cu_B_. Lacking copper, cytochromes *bd*-I and *bd*-II each contain a ubiquinol/menaquinol binding site and three hemes (*b*_558_, *b*_595_, and *d*). Heme *d* is the site in which the tight binding of O_2_ and its subsequent reduction to 2H_2_O occurs [21].

Unlike cytochrome *bo*_3_, cytochrome *bd*-I shows a high resistance to inhibition by cyanide, NO, peroxynitrite, H_2_S, H_2_O_2_, and CO [21]. Cytochrome *bd*-II is much less understood than cytochrome *bd*-I. The *bd*-II enzyme was shown to be resistant to cyanide, H_2_S, and H_2_O_2_, but, in contrast to *bd-*I, sensitive to CO [21].

The *bd*-type terminal oxidases are often found in pathogenic bacteria, likely because they contribute to resistance against oxidative, nitrosative, and other stresses induced by toxic small molecules and antibiotics [21,22,23,24]. Since the human genome does not encode bacterial *bd*-type cytochromes, these enzymes represent promising therapeutic targets for new antimicrobials [9]. The development of novel antimicrobial drugs is an urgent global health priority [25,26]. Therefore, interest in studying the *bd*-type cytochromes has grown significantly in recent years [27,28,29,30,31,32,33,34,35,36,37,38,39,40,41,42,43,44,45,46,47,48,49,50,51,52,53,54].

*E. coli* serves as a ubiquitous and permanent resident within the guts of humans and homeothermic animals, where it actively consumes available O_2_. Remarkably, ammonia levels can reach the tens-of-millimolar range in specific segments of the intestine [55,56,57]. However, how such high ammonia concentrations affect respiration in *E. coli* and other enterobacteria remains poorly understood. We previously showed that at pH 8.3, ammonia inhibits O_2_ consumption in the isolated cytochrome *bo*_3_, whereas it accelerates consumption in both the isolated cytochrome *bd*-I and *bd*-I-only cells [58]. In this study, we extended our previous research [58] to address the following questions: How do high concentrations of ammonia affect *E. coli* cell growth? How does this relate to the previously identified differences in ammonia’s effect on cytochrome *bo*_3_ and *bd*-I activity? What is the effect of ammonia on the O_2_ consumption by cytochrome *bd*-II, and how does this influence the growth of *E. coli* cells relying exclusively on this terminal oxidase?

## 2. Materials and Methods

### 2.1. Reagents, E. coli Strains and Growth Conditions

Ammonium sulfate and Tris Base were purchased from Carlo Erba (Carlo Erba Reagents S.r.l., Milan, Italy) and Fisher BioReagents (Thermo Fisher Scientific, Pittsburgh, PA, USA), respectively. Other chemicals were purchased from Sigma-Aldrich (Sigma-Aldrich, St. Louis, MO, USA) We used the following *E. coli* respiratory mutant strains derived from the K-12 derivative MG1655 (RKP5416): TBE025 (MG1655 Δ*cydB nuoB appB::kan*), TBE026 (MG1655 Δ*cydB nuoB cyoB::kan*) and TBE037 (MG1655 Δ*appB nuoB cyoB::kan*). The strains express cytochrome *bo*_3_, cytochrome *bd*-II or cytochrome *bd*-I as the only terminal respiratory oxidase, respectively [59]. They were kindly given by Alex Ter Beek and Joost Teixeira de Mattos (University of Amsterdam). *E. coli* cells were grown in Luria–Bertani (LB) broth medium supplemented with 30 μg/mL kanamycin, at 37 °C and 200 rpm. For growth studies, the cells were grown in 50 mL Falcon tubes, in 5 mL of air-equilibrated LB for 90 min at 37 °C and then treated with either (NH_4_)_2_SO_4_ as an experimental reagent of the ammonia source or K_2_SO_4_ as a control to yield a final concentration of the salt of 27 mM. This concentration was selected based on findings that at this concentration, the effect of ammonia on the enzymatic O_2_ reductase activity was maximal or close to maximal [58]. At the same time, the contribution of nonspecific ionic strength effects remained substantially lower than at higher ammonium sulfate concentrations. Cell growth was then monitored via a standard procedure with the use of optical density measurements in an Eppendorf BioSpectrometer (Eppendorf, Hamburg, Germany) basic at a wavelength of 600 nm every 30 min. When the OD_600_ exceeded 1.0, *E. coli* cultures were diluted before reading.

For microcalorimetry studies, once the cells cultured in LB at 37 °C reached the OD_600_ of 0.1–0.3, they were diluted approximately to the OD_600_ of 0.001 in LB (pH 8.3), containing 27 mM (NH_4_)_2_SO_4_ (as an experimental reagent of the ammonia source), or 27 mM K_2_SO_4_ (as a control). The samples were transferred to the isothermal microcalorimeter plate, equipped with titanium vials and plastic inserts. The vials were sealed and placed in a calScreener^®^ isothermal microcalorimeter (Symcel AB, Solna, Sweden) in accordance with the manufacturer’s guidelines. Kinetic heat flow was monitored using the calView^TM^ 2.0 software (Symcel AB), which recorded heat flow curves. The maximum heat flow was calculated by subtracting the initial heat flux value from the maximum peak value. The measurements were carried out at 37 °C.

### 2.2. Preparation of E. coli Membranes Containing Cytochrome bd-II as the Only Terminal Respiratory Oxidase

To do this, mutant strain TBE026 expressing cytochrome *bd*-II as the only terminal oxidase was used. *E. coli* culture was cultivated in LB medium at 37 °C until an OD_600_ of approximately 2 was reached. Then, the cells were sedimented by centrifugation (10,000 rpm, 10 min, 4 °C) and washed twice in a medium containing 20 mM Tris, 0.5 mM EDTA and 5 mM MgSO_4_, pH 8.3. The cells were resuspended in the same medium containing 1 mg/mL lysozyme and incubated for two hours on ice. The resulting cell suspension was treated with RNase and DNase. Then, the cells were lysed by sonication. Cell debris was removed by centrifugation (15,000 rpm, 10 min, 4 °C). Membrane fractions were collected and stored at −80 °C. Protein content was quantified using the Bradford protein assay with bovine serum albumin as a standard.

### 2.3. Isolation of Cytochrome bd-II

Cytochrome *bd*-II was isolated as described previously [11]. The thawed *bd*-II-only membranes were solubilized with sucrose monolaurate (1.8% final concentration). The solubilized membrane suspension was centrifuged (160,000 *g*, 60 min, 4 °C), and the pellet was discarded. The supernatant was loaded on a DEAE Sepharose Fast Flow anion exchange column equilibrated with a buffer containing 50 mM potassium phosphate, 25 mM KCl, 5 mM EDTA, and 0.1% sucrose monolaurate, pH 6.5. The elution was performed at 4 °C by using a linear KCl gradient of 25–470 mM. The fractions with an absorbance ratio of A_412_/A_280_ higher than 0.7 were pooled and concentrated. Cytochrome *bd*-II sample aliquots were subjected to rapid freezing in liquid nitrogen and then stored at −80 °C. The cytochrome *bd*-II concentration was determined from the difference absorption spectrum (sodium dithionite-reduced enzyme minus air-oxidized enzyme) using Δ*ε*_628–607_ = 10.8 mM^−1^ cm^−1^ [60].

### 2.4. O_2_ Consumption Measurements and Assay Conditions

The O_2_ consumption of cytochrome *bd-*II-containing *E. coli* cells, membranes and isolated *bd-*II enzyme was measured using a high-resolution respirometer (NextGen-O2k all-in-one, Oroboros Instrument, Innsbruck, Austria) equipped with two 1.5 mL chambers. Measurements were carried out at 25 °C in 100 mM Tris-HCl (pH 8.3) or 100 mM potassium phosphate (pH 7.0) buffer. In the case of membranes and isolated enzyme, an excess of the reducing agents, 10 mM DTT and 0.25 mM Q_1_, was added to sustain the O_2_-reductase activity of cytochrome *bd*-II. In the case of isolated enzyme, the buffer was also supplemented with 0.1 mM EDTA, 2.5 μg/mL catalase and 0.02% dodecyl-β-D-maltoside. The pH of the stock solutions of (NH_4_)_2_SO_4_ and K_2_SO_4_ was adjusted to the desired values (8.3 or 7.0).

### 2.5. Data Analysis

Data analysis was performed using Origin (OriginLab Corporation, Northampton, MA, USA). The ammonia titration data obtained in oxygraphic experiments at pH 8.3 were fitted to the standard hyperbolic equation y = *A*_max_·x/(*K*_d_*_app_* + x) using a built-in approximation function (‘Hyperbola function’) in the ‘Advanced Fitting tool’ in the Origin program. *A*_max_ and *K*_d_*_app_* parameters were allowed to vary. *K*_d_*_app_* is an apparent dissociation constant; *A*_max_ is a theoretical maximum percent activity (O_2_ consumption rate). To take into account a possible effect of the high ionic strength on the cytochrome *bd*-II activity, K_2_SO_4_ at the same concentration was added to the sample instead of (NH_4_)_2_SO_4_ (as an experimental reagent of the ammonia source) for each condition as a control.

## 3. Results

### 3.1. Effect of Ammonia on E. coli Cell Growth

**Figure 1:**
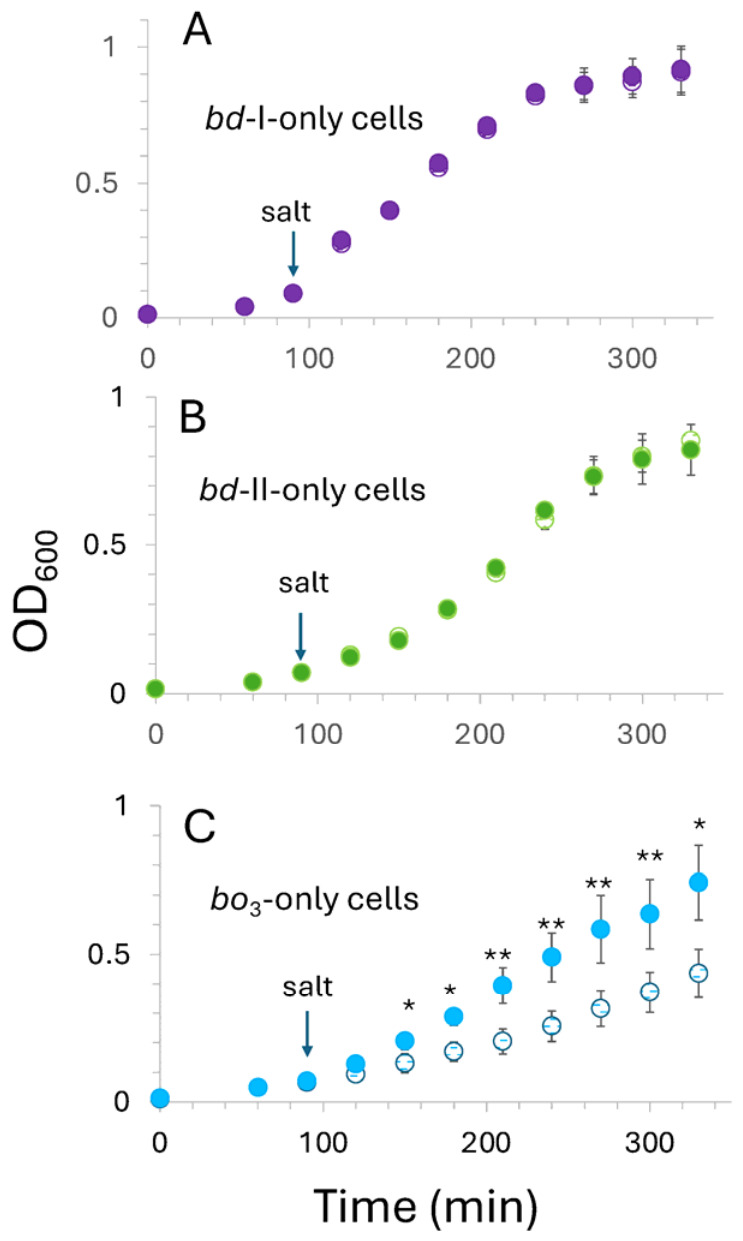
Effect of ammonia on *E. coli* cell growth at pH 8.3. Cell growth of *E. coli* mutant strains expressing either cytochrome *bd*-I (**A**), cytochrome *bd*-II (**B**) or cytochrome *bo*_3_ (**C**) as the sole terminal oxidase was monitored in the presence of either 5.4 mM NH_3_ (supplied as 27 mM (NH_4_)_2_SO_4_; ‘open symbols’) or 27 mM K_2_SO_4_ (ionic strength control; ‘closed symbols’). The arrow indicates the time (90 min) at which the salt was added to a growing cell culture. Data are presented as the mean ± standard deviations from three independent experiments. Asterisks indicate statistically significant differences between the NH_3_ and K_2_SO_4_ treatments in the mutant strain (*, *p* < 0.05; **, *p* < 0.01).

The first objective of this study was to determine whether any of the three terminal oxidases in *E. coli* could promote cell growth in the presence of a high concentration of ammonia. To do this, we examined the growth of three different mutant strains, each expressing only one of three respiratory enzymes, at pH 8.3 in the presence of either 5.4 mM NH_3_ (27 mM (NH_4_)_2_SO_4_) or 27 mM K_2_SO_4_ as a control. Figure 1A shows that the addition of 5.4 mM NH_3_ (27 mM (NH_4_)_2_SO_4_) to cytochrome *bd*-I-only cells has no effect on their growth compared to the control with 27 mM K_2_SO_4_. The same is observed with *E. coli* cells containing cytochrome *bd*-II as the sole terminal oxidase (Figure 1B). In contrast, the growth of the strain expressing cytochrome *bo*_3_ as the only respiratory enzyme is significantly impaired over the same time window after the addition of 5.4 mM NH_3_ (27 mM (NH_4_)_2_SO_4_), compared to the control with 27 mM K_2_SO_4_ (Figure 1C). Thus, we can conclude that under these conditions, both *bd*-type oxidases, *bd*-I and *bd*-II, promote *E. coli* growth in the presence of a high concentration of ammonia, whereas the heme-copper *bo*_3_ oxidase does not.

**Figure 2:**
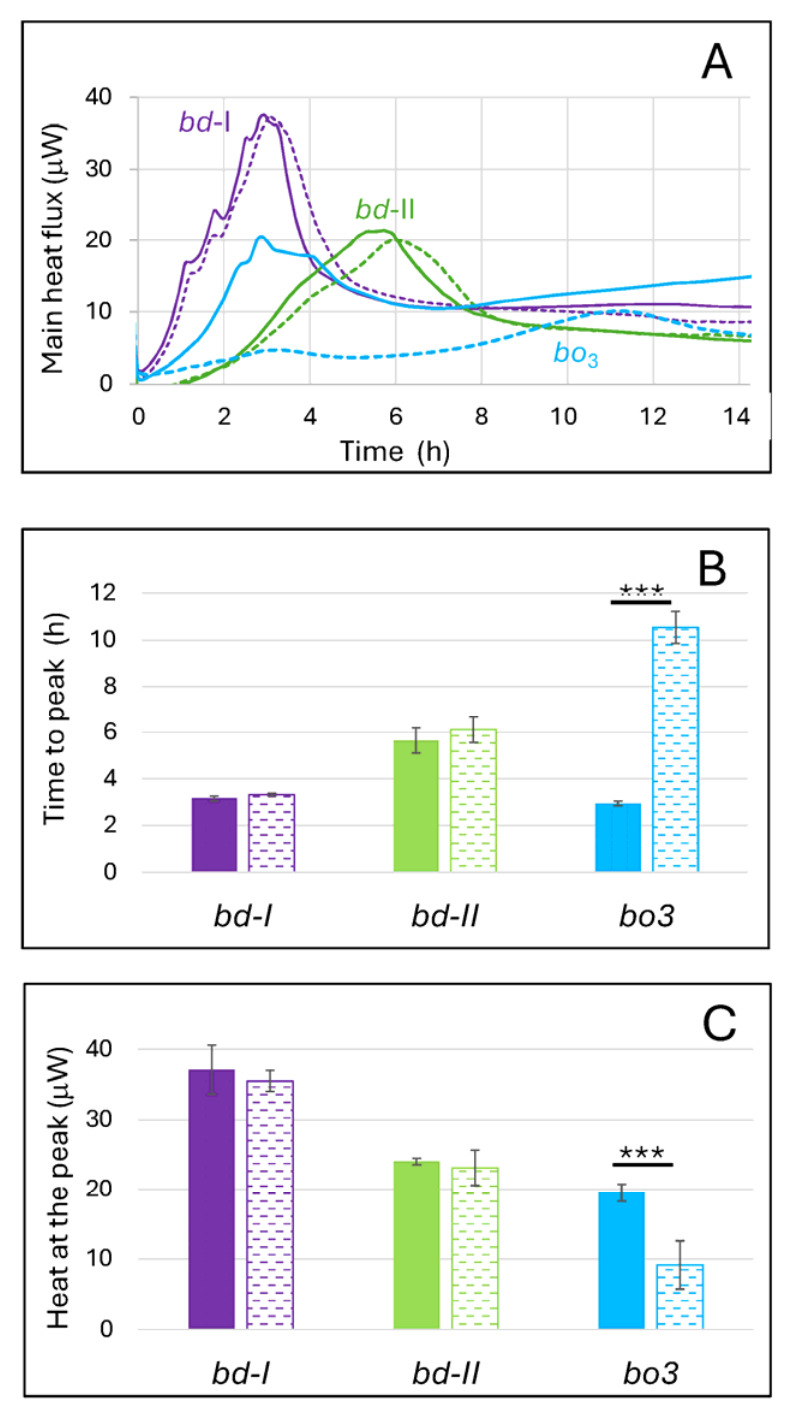
Effect of ammonia on *E. coli* metabolic activity at pH 8.3. (**A**) Thermogram showing metabolic activity of *E. coli* mutant strains expressing either cytochrome *bd*-I (violet), cytochrome *bd*-II (green), or cytochrome *bo*_3_ (light blue) as the sole terminal oxidase in the presence of either 27 mM K_2_SO_4_ (ionic strength control; solid line) or 5.4 mM NH_3_ (supplied as 27 mM (NH_4_)_2_SO_4_; dashed line). (**B**) Time required to reach the maximum heat-flow peak (exponential growth phase) for the three different *E. coli* mutant strains in the presence of 27 mM K_2_SO_4_ (solid bar) or 5.4 mM NH_3_ (supplied as 27 mM (NH_4_)_2_SO_4_, dashed bar). (**C**) Maximum heat flow (μW) produced by the *E. coli* mutant strains in the presence of 27 mM K_2_SO_4_ (solid bar) or 5.4 mM NH_3_ (supplied as 27 mM (NH_4_)_2_SO_4_; dashed bar). Data are presented as the mean ± standard deviations from four independent experiments. Asterisks (***) indicate statistically significant differences between the NH_3_ and K_2_SO_4_ treatments in the mutant strain (*p* < 0.001).

To measure the *E. coli* metabolic activity in the presence of a high concentration of ammonia at pH 8.3, we used isothermal microcalorimetry. This method detects the heat produced by microorganisms during their physiological activity. Figure 2A shows a typical real-time thermogram displaying heat flow produced by the *bd*-I-, *bd*-II- and *bo_3_*-only expressing strains obtained with 5.4 mM NH_3_ (27 mM (NH_4_)_2_SO_4_) or 27 mM K_2_SO_4_ in the culture media. The three *E. coli* mutant strains exhibit distinct thermal profiles, as is to be expected given the expression of diverse oxidases that differ in their affinity for O_2_, bioenergetic efficiency and levels of oxidase activity.

In the strains containing only a *bd*-type cytochrome, the heat profiles measured in the presence of K_2_SO_4_ and ammonia are comparable; in contrast, in the case of the *bo*_3_-only strain, significant differences are observed. First, ammonia appears to limit the *bo*_3_ oxidase activity and prolong the initial exponential phase, shifting the maximum peak—that is, the maximum metabolic activity observed in the presence of K_2_SO_4_—by approximately 9 h, as reported also in Figure 2B. Furthermore, there are clear differences in signal amplitude. Ammonia reduces by the half the height of the maximum peak registered with K_2_SO_4_ in the medium, i.e., the heat flux (Figure 2A,C), indicating a substantially reduced metabolic activity.

### 3.2. Effect of Ammonia on O_2_ Consumption of E. coli Cytochrome bd-II in Different Environments

The second objective of this study was to examine the influence of ammonia on the O_2_-reductase activity of cytochrome *bd*-II in different environments: cells, membranes or isolated in detergent micelles.

#### 3.2.1. Cells

**Figure 3:**
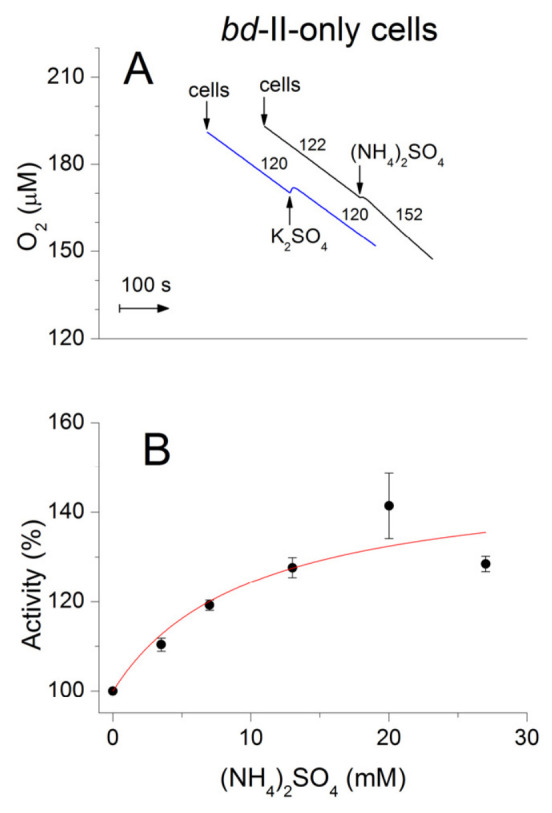
Effect of ammonia on O_2_ consumption by cytochrome *bd-*II-containing *E. coli* cells at pH 8.3. (**A**) Representative O_2_ consumption traces. O_2_ consumption rates (nM O_2_·s^−1^), measured before and after the addition of either 5.4 mM NH_3_ (supplied as 27 mM (NH_4_)_2_SO_4_) or 27 mM K_2_SO_4_ (ionic strength control) to a 350 μL cell suspension (OD_600_ = 1.4) are indicated next to each trace. Data are representative of three independent experiments (*n* = 3). (**B**) Dependence of the O_2_ consumption rate on ammonia concentration. NH_3_ concentrations of 0, 0.7, 1.4, 2.6, 4.0, and 5.4 mM correspond to the addition of 0, 3.5, 7, 13, 20, and 27 mM (NH_4_)_2_SO_4_ respectively, which served as the ammonia source. Values are expressed with reference to the activity measured before addition of ammonia taken as 100%. To take into account the effect of increasing ionic strength on the rate of O_2_ consumption of cytochrome *bd-*II, a value measured in the presence of K_2_SO_4_ was subtracted from that in the presence of ammonia at the same concentration of the salt. Experimental data (*filled circles*) are shown together with their best fit (*solid line*) to the hyperbolic equation (see Materials and Methods), yielding a maximum activity value *A*_max_ of 148.5 ± 14.7%, and *K*_d_*_app_* of 2.0 ± 1.5 mM NH_3_ (9.9 ± 7.5 mM (NH_4_)_2_SO_4_) (mean ± standard deviation, *n* = 3).

We found that at pH 8.3, the addition of up to 5.4 mM NH_3_ (27 mM (NH_4_)_2_SO_4_) to *E. coli* intact cells expressing cytochrome *bd-*II as the sole respiratory oxidase accelerates their O_2_ consumption (Figure 3A,B). No external respiratory substrate was added as the O_2_ consumption of the *E. coli* cells was sustained by endogenous reductants. The highest increase in the O_2_ consumption rate (141.4 ± 7.2%) was observed at 4 mM NH_3_ (20 mM (NH_4_)_2_SO_4_) (Figure 3B). Virtually no change in the O_2_ consumption rate was detected when K_2_SO_4_ was added at the same salt concentration as a control (Figure 3A). At pH 7, the effect of (NH_4_)_2_SO_4_, an experimental reagent of the ammonia source, on the O_2_ consumption of the cell suspension is much less pronounced (Supplementary Figure S1A). This indicates that the primary effector molecule is ammonia (NH_3_). Analysis of the concentration dependence plot shown in Figure 3B (percent activation versus ammonia ((NH_4_)_2_SO_4_) concentration at pH 8.3) gives a maximum O_2_ consumption rate *A*_max_ of 148.5 ± 14.7% and an apparent dissociation constant *K*_d_*_app_* of 2.0 ± 1.5 mM NH_3_ (9.9 ± 7.5 mM (NH_4_)_2_SO_4_).

#### 3.2.2. Membranes

**Figure 4:**
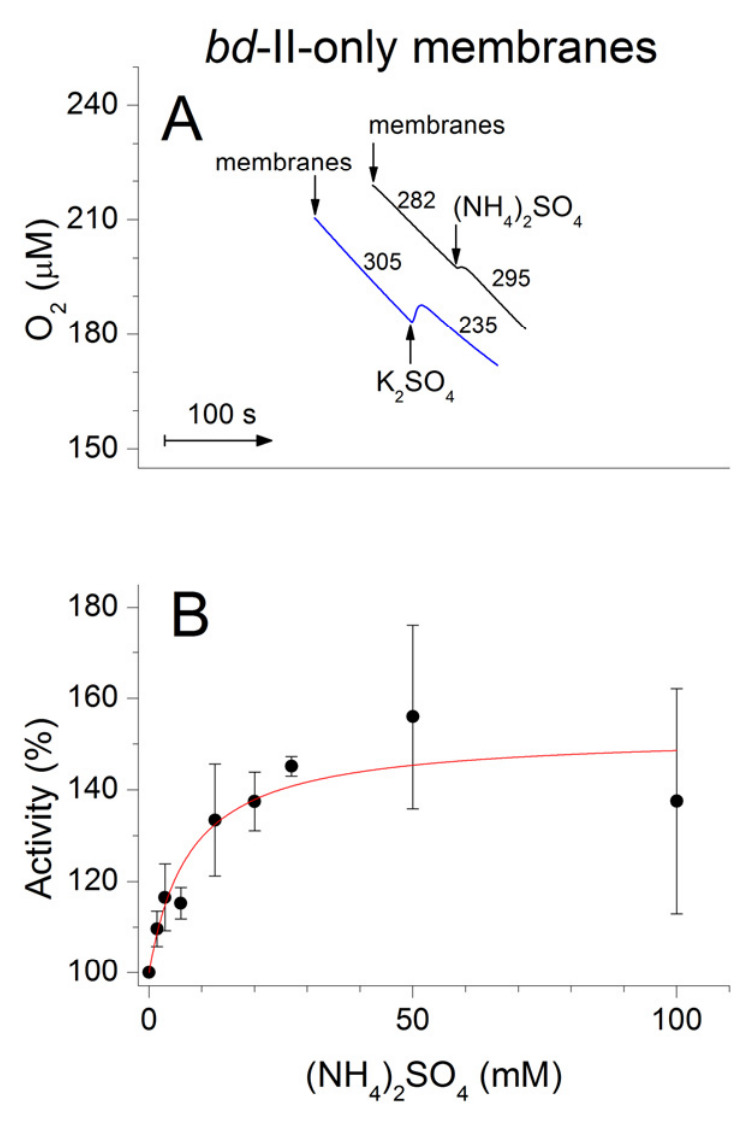
Effect of ammonia on O_2_ consumption by cytochrome *bd-*II-containing *E. coli* membranes at pH 8.3. (**A**) Representative O_2_ consumption traces. O_2_ consumption rates (nM O_2_·s^−1^), measured before and after the addition of either 10 mM NH_3_ (supplied as 50 mM (NH_4_)_2_SO_4_) or 50 mM K_2_SO_4_ (ionic strength control) are indicated next to each trace. Data are representative of three independent experiments (*n* = 3). (**B**) Dependence of the O_2_ consumption rate on ammonia concentration. NH_3_ concentrations of 0, 0.3, 0.6, 1.2, 2.5, 4, 5.4, 10, and 20 mM correspond to the addition of 0, 1.5, 3, 6, 12.5, 20, 27, 50, and 100 mM (NH_4_)_2_SO_4_ respectively, which served as the ammonia source. Values are expressed with reference to the activity measured before addition of ammonia taken as 100%. To take into account the effect of increasing ionic strength on the rate of O_2_ consumption of cytochrome *bd-*II, a value measured in the presence of K_2_SO_4_ was subtracted from that in the presence of ammonia at the same concentration of the salt. Experimental data (*filled circles*) are shown together with their best fit (*solid line*) to the hyperbolic equation (see Materials and Methods), yielding a maximum activity value *A*_max_ of 152.3 ± 6.3%, and *K*_d_*_app_* of 1.5 ± 0.7 mM NH_3_ (7.6 ± 3.3 mM (NH_4_)_2_SO_4_) (mean ± standard deviation, *n* = 3). The enzyme activity is sustained by 10 mM DTT and 0.25 mM Q_1_. Membranes, 80 μg protein/mL.

We also tested how the addition of ammonia at high concentrations affects the O_2_ consumption of cytochrome *bd-*II-containing *E. coli* membranes. Consistent with data on the intact cells, treatment of the membranes with ammonia increases their O_2_ reductase activity sustained by a DTT and Q_1_ pair (Figure 4A,B). The O_2_ consumption rate at pH 8.3 increases with an increasing ammonia concentration, and the largest increase (156.0 ± 20.1%) is observed at 10.09 mM NH_3_ (50 mM (NH_4_)_2_SO_4_) (Figure 4B). On the contrary, the addition of K_2_SO_4_ at the same salt concentration as the control decreases the respiratory activity of the membranes (Figure 4A). This is likely due to the effect of the high ionic strength [61,62]. As with the cells, at pH 7, acceleration in O_2_ consumption of the membranes induced by (NH_4_)_2_SO_4_, an experimental reagent of the ammonia source, was less notable (Supplementary Figure S1B). Analysis of the concentration dependence plot (Figure 4B) at pH 8.3 yields an *A*_max_ of 152.3 ± 6.3% and *K*_d_*_app_* of 1.5 ± 0.7 mM NH_3_ (7.6 ± 3.3 mM (NH_4_)_2_SO_4_) (Figure 4B).

#### 3.2.3. Isolated Enzyme

**Figure 5:**
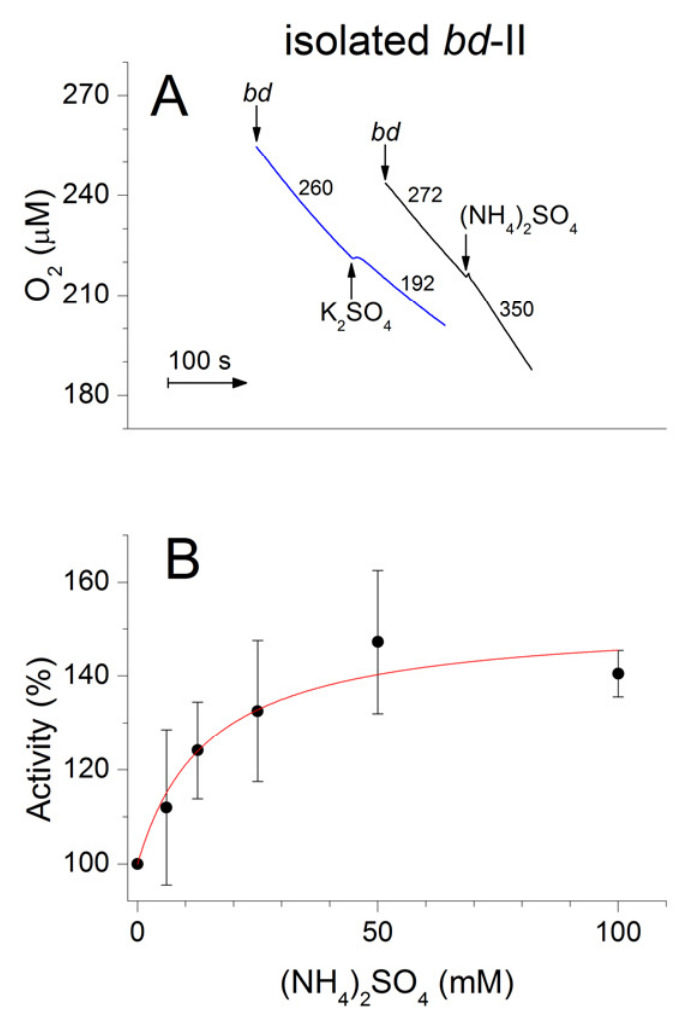
Effect of ammonia on O_2_ consumption by the isolated cytochrome *bd-*II of *E. coli* at pH 8.3. (**A**) Representative O_2_ consumption traces. O_2_ consumption rates (nM O_2_·s^−1^), measured before and after the addition of either 10 mM NH_3_ (supplied as 50 mM (NH_4_)_2_SO_4_) or 50 mM K_2_SO_4_ (ionic strength control) are indicated next to each trace. Data are representative of three independent experiments (*n* = 3). (**B**) Dependence of the O_2_ consumption rate on ammonia concentration. NH_3_ concentrations of 0, 1.2, 2.5, 5, 10, and 20 mM correspond to the addition of 0, 6, 12.5, 25, 50, and 100 mM (NH_4_)_2_SO_4_ respectively, which served as the ammonia source. Values are expressed with reference to the activity measured before addition of ammonia taken as 100%. To take into account the effect of increasing ionic strength on the rate of O_2_ consumption of cytochrome *bd-*II, a value measured in the presence of K_2_SO_4_ was subtracted from that in the presence of ammonia at the same concentration of the salt. Experimental data (*filled circles*) are shown together with their best fit (*solid line*) to the hyperbolic equation (see Materials and Methods), yielding a maximum activity value *A*_max_ of 152.2 ± 6.2%, and *K*_d_*_app_* of 2.9 ± 1.1 mM NH_3_ (14.7 ± 5.6 mM (NH_4_)_2_SO_4_) (mean ± standard deviation, *n* = 3). The enzyme activity is sustained by 10 mM DTT and 0.25 mM Q_1_. Cytochrome *bd-*II, 3.5 nM.

Finally, we studied the influence of ammonia on the O_2_ consumption of the isolated cytochrome *bd-*II. The results are fully consistent with those obtained with the cells and membranes with cytochrome *bd-*II as the only terminal oxidase. The addition of ammonia to the isolated enzyme at pH 8.3 in the presence of O_2_ and DTT/Q_1_ increases its O_2_ reductase activity. In contrast, the treatment of cytochrome *bd-*II with K_2_SO_4_ decreases the enzyme activity due to the increase in ionic strength (Figure 5A,B). The fastest acceleration of the O_2_ consumption rate (147.2 ± 15.2%) was observed at 10.09 mM NH_3_ (50 mM (NH_4_)_2_SO_4_) (Figure 5B). At pH 7, the addition of (NH_4_)_2_SO_4_, an experimental reagent of the ammonia source, to the enzyme does not lead to a marked increase in the O_2_ consumption rate (Supplementary Figure S1C). Analysis of the concentration dependence plot (Figure 5B) at pH 8.3 gives an *A*_max_ of 152.2 ± 6.2%, and *K*_d_*_app_* of 2.9 ± 1.1 mM NH_3_ (14.7 ± 5.6 mM (NH_4_)_2_SO_4_).

## 4. Discussion

### 4.1. Modulation of E. coli Growth by Terminal Oxidase: Physiological Implications Under Ammonia Stress

Ammonia has long been considered just a waste product of protein catabolism. Recently, the traditional concept has changed. Ammonia can now be qualified as a new member of the family of endogenously produced gasotransmitters, which also includes the classic triad—nitric oxide, carbon monoxide and hydrogen sulfide. These are freely permeable, small and reactive gaseous signaling molecules implicated in a number of physiological and pathophysiological processes [63]. For instance, ammonia acts as a critical signaling molecule, promoting the progression of malignant tumors via regulation of metabolic remodeling, epigenetic modifications, and tumor metastasis [64,65]. Accumulating data suggest that ammonia plays a signaling role in cultured astrocytes of rats [66,67] and mice [68]. It can also act through glial GABAergic signaling to regulate neuromuscular transmission in the enteric nervous system [56]. In addition, ammonia serves as a potent infochemical in interbacterial communication. By triggering oxidative stress responses and enhancing antibiotic tolerance, it contributes to the antimicrobial resistance mechanisms of bacteria [69].

Toxic levels of ammonia can lead to neurological dysfunction. For this reason, in healthy adults, blood ammonia concentrations are tightly maintained below 50 μM [70]. In contrast, endogenous NH_3_ production in the adult human gastrointestinal tract yields approximately 4 to 10 g daily [71]. In fact, total ammonia concentrations within the intestinal lumen are typically three orders of magnitude higher than those found in blood [57], suggesting that bacterial tolerance to ammonia may play a role in shaping the human gut microbiota. Using a model of conscious Large White pig with a canula implanted into the proximal colon, Eklou-Lawson et al. showed that the peak ammonia concentration occurs within the large intestinal lumen, reaching an estimated 27.2 ± 17.5 mM [57]. Earlier work reported a broader range of luminal ammonia concentrations within the colon, ranging from 4 to 70 mM [55,56]. The luminal pH varies significantly throughout the intestinal tract, depending on the specific anatomical segment, dietary intake, and the biogeographical distribution of the microbiota, occasionally reaching alkaline levels. In the distal ileum, for instance, the median pH exhibits a distinctly basic value of 8.1 [72].

We have recently begun to study the interaction of the *E. coli* terminal oxidases *bd*-I and *bo*_3_ with ammonia [58]. We found that the O_2_ consumption of the isolated cytochrome *bo*_3_ is partially inhibited by ammonia at pH 8.3 [58]. In contrast, under identical conditions, following ammonia addition, the O_2_ consumption accelerates both in isolated cytochrome *bd*-I and in *bd*-I-only containing cell cultures [58]. The present work is a continuation of that study. We intended to answer the following main questions. First, whether the previously reported data [58] are relevant to *E. coli* physiology. Specifically, how the *E. coli* cells grow in the presence of a high ammonia concentration and if this correlates with the effect of ammonia on the activity of the *bd*-I and *bo*_3_ enzymes reported in [58]. To our knowledge, no previous studies have investigated the growth issue. Second, how the third *E. coli* terminal oxidase, cytochrome *bd*-II, interacts with ammonia. Specifically, how ammonia influences the growth of *bd*-II-only cells, and the O_2_ consumption across different levels of biological complexity—ranging from whole *bd*-II-only cells to isolated *bd*-II-only membranes and the purified enzyme.

We received answers to these questions. Using two different methods to monitor the cell growth of three different respiratory mutants, each possessing only one terminal oxidase, we showed that when *E. coli* cells rely on either *bd*-I or *bd*-II to sustain aerobic respiration, the growth is unaffected by 5.4 mM NH_3_ (27 mM (NH_4_)_2_SO_4_) (Figure 1A,B and Figure 2). Consistently, the catalytic activity of both *bd*-I in cells and as an isolated enzyme [58], and *bd*-II in different environments, including cells, membranes and as isolated enzyme, is not inhibited but significantly increased after the addition of ammonia (Figure 3, Figure 4 and Figure 5). On the contrary, when the *E. coli* cells rely only on *bo*_3_ to sustain respiration, the growth is substantially inhibited (Figure 1C) and the metabolic activity severely reduced (Figure 2). Consistently, the catalytic activity of the purified cytochrome *bo*_3_ is also inhibited [58]. Thus, the present study suggests that both cytochrome *bd*-I and cytochrome *bd*-II promote the growth of *E. coli* and possibly other bacteria in ammonia-rich environments. Although cytochrome *bo*_3_, as a proton pump, is twice as energetically efficient as *bd*-I and *bd*-II [10,11], it did not appear to be capable of doing so. These findings also support the idea that *E. coli* respiration links cellular energy pathways into an overall metabolic response modulated by environmental factors such as ammonia. Measuring respiration rates is therefore an excellent way of monitoring the adaptive responses of *E. coli* and other bacteria and their health, as evidenced by the data obtained via microcalorimetry.

### 4.2. Plausible Mechanism for Acceleration of O_2_ Consumption of Cytochrome bd-II by Ammonia

As noted above, at pH 8.3, the O_2_ consumption of cytochrome *bd-*II is not inhibited by millimolar concentrations of ammonia. On the contrary, the addition of ammonia accelerates the reaction. The greatest increase in the O_2_ consumption rate observed was 141.4 ± 7.2%, 156.0 ± 20.1%, and 147.2 ± 15.2% for *bd-*II-only cells, membranes, and the isolated detergent-solubilized enzyme, respectively (Figure 3, Figure 4 and Figure 5). Evaluating this effect across different environments of the oxidase was crucial, as variations in the protein environments can significantly alter *bd*-type cytochrome sensitivity to a ligand, as recently demonstrated for CO [73,74].

Both the present study and our previous work [58] support the conclusion that NH_3_, rather than NH_4_^+^, is the main active species affecting activity of the *E. coli* terminal oxidases. In this study, the conclusion is based on the observation that the stimulation of cytochrome *bd*-II activity by (NH_4_)_2_SO_4_ is markedly greater at pH 8.3 than at pH 7.0.

The NH_4_^+^/NH_3_ equilibrium is strongly pH-dependent. Therefore, for a given total concentration of ammonium ((NH_4_)_2_SO_4_), the concentrations of NH_4_^+^ and free NH_3_ can be estimated using the Henderson–Hasselbalch equation (p*K*_a_ = 9.25 at 25 °C). For example, at a total concentration of 50 mM (NH_4_)_2_SO_4_, the calculated concentrations at pH 7.0 are approximately 99.44 mM NH_4_^+^ and 0.56 mM NH_3_, whereas at pH 8.3, they are approximately 89.91 mM NH_4_^+^ and 10.09 mM NH_3_. Thus, increasing the pH from 7.0 to 8.3 results in an approximately 18-fold increase in free NH_3_ concentration, while the NH_4_^+^ concentration changes by less than 10%. The pronounced increase in cytochrome *bd*-II activation therefore correlates with the increase in NH_3_ rather than with the relatively small change in NH_4_^+^ concentration.

**Figure 6:**
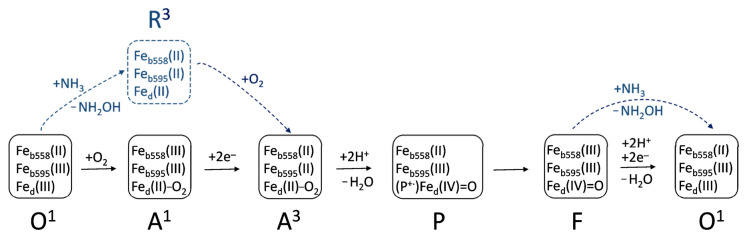
Proposed effect of ammonia on the catalytic cycle of *E. coli* cytochrome *bd-*II. Ammonia (NH_3_) is hypothesized to accelerate enzyme turnover by reducing the catalytic intermediates F (*b*_558_^3+^*b*_595_^3+^*d*^4+^ = O^2−^) and O^1^ (*b*_558_^2+^*b*_595_^3+^*d*^3+^), while being oxidized to hydroxylamine (NH_2_OH). During the reaction of NH_3_ with the F intermediate, one electron from NH_3_ is transferred to Fe^4+^ = O^2−^ of heme *d*, while the second electron is transferred to Fe^3+^ of heme *b*_558_. Consequently, Fe^4+^ = O^2−^ of heme *d* is reduced to Fe^3+^, and Fe^3+^ of heme *b*_558_ is reduced to Fe^2+^, whereas Fe^3+^ of heme *b*_595_ does not change its redox state. This reaction generates the O^1^ intermediate. The oxygen atom incorporated into NH_2_OH may originate from the ferryl oxygen (Fe^4+^ = O^2−^) of heme *d*. In the reaction of NH_3_ with the O^1^ intermediate, one electron is transferred to Fe^3+^ of heme *d*, and the second electron to Fe^3+^ of heme *b*_595_. As a result, both heme *d* and heme *b*_595_ are reduced to the Fe^2+^ state, yielding the R^3^ species (*b*_558_^2+^*b*_595_^2+^*d*^2+^). If the O^1^ intermediate contains a hydroxide ligand coordinated to Fe^3+^ of heme *d*, the oxygen atom incorporated into NH_2_OH may originate from Fe^3+^-OH in this reaction. Unlike F and O^1^, the R^3^ species is not part of the normal catalytic cycle. However, it binds O_2_ more rapidly than the O^1^ intermediate, thereby providing a potential explanation for the observed stimulation of enzyme activity by ammonia.

Figure 6 shows the possible effect of ammonia on the catalytic cycle of cytochrome *bd-*II. In the normal catalytic cycle, O_2_ binds to the O^1^ species (*b*_558_^2+^*b*_595_^3+^*d*^3+^), yielding the A^1^ species (*b*_558_^3+^*b*_595_^3+^*d*^2+^–O_2_). A^1^ accepts two electrons from the first quinol molecule, giving the A^3^ species (*b*_558_^2+^*b*_595_^2+^*d*^2+^–O_2_). A^3^ is converted into the P species first discovered by Belevich et al. [75]. Paulus et al. [76] suggested that during the A^3^→P transition, O–O bond cleavage occurs and therefore, in the P species, heme *d* is in the ferryl state, with an π-cation radical on the porphyrin ring (*b*_558_^2+^*b*_595_^3+^*d*^4+^ = O^2−^ (P^+•^)). P is then converted into the F species (*b*_558_^3+^*b*_595_^3+^*d*^4+^ = O^2−^). Finally, F accepts two electrons from the second quinol molecule, yielding O^1^and one catalytic cycle is closed.

We hypothesize that NH_3_ can donate two electrons to the F and O^1^ species. In these reactions, NH_3_ is oxidized to NH_2_OH. Earlier, we showed that in the *E. coli* cytochrome *bd-*I, in a steady state, the F species is a highly populated catalytic intermediate (~40%) [77]. Ferryl species in heme proteins have a very high redox potential (~+1 V) [78] and thus are a strong oxidant. Therefore, we suggest that NH_3_ can react with F by producing O^1^, thereby resulting in the acceleration of the enzyme activity.

As for a second possible reaction, the oxidation of NH_3_ to NH_2_OH would be accompanied by the conversion of O^1^ to R^3^. It has to be noted that in the absence of ammonia, R^3^ is likely not to participate in the catalytic cycle of cytochrome *bd*. We suggest that R^3^ binds O_2_ faster than O^1^, thereby increasing the overall rate of O_2_ consumption. This suggestion is consistent with an earlier observation that the binding rate of O_2_ with the *Azotobacter vinelandii* cytochrome *bd* in the fully reduced state depends linearly on the O_2_ concentration but is hyperbolic when the enzyme is in the one-electron-reduced state (see Figure 3C in Belevich et al. [79]). It had been proposed that O_2_ binding to heme *d* requires an open (‘activated’) state of cytochrome *bd*. In the fully reduced cytochrome *bd*, the entire enzyme population is in the open state, while the one-electron-reduced enzyme population represents a mixture of the open and closed states [79].

The proposed reactions of NH_3_ with the catalytic intermediates F and O^1^ are described in greater detail in the caption of Figure 6.

It should be emphasized that the proposed mechanism by which cytochrome *bd*-II oxidizes ammonia is speculative and requires further experimental validation. Such validation could include direct detection of the reaction product (NH_2_OH), spectroscopic characterization of the catalytic intermediates, isotope-labeling experiments to determine the source of the oxygen atom in NH_2_OH, or kinetic modeling of the proposed reaction pathway. These investigations were beyond the scope of the present study.

## 5. Conclusions

Differences in respiratory oxidase resistance to various environmental stresses may have profound impacts on microbial physiology, contributing to the success of bacterial colonization in diverse ecological niches and to competition between species. Our findings suggest that *E. coli* and possibly other bacteria can evade ammonia-mediated respiratory inhibition by modulating the terminal segment of their electron transfer chains. Such a metabolic adaptation can sustain microbial growth and energy production in ammonia-dense niches, including animal gastrointestinal tracts, manure storage, some aquatic sediments, fertilizer-treated agricultural soils and wastewater bioreactors. Accordingly, these results may have biomedical implications, as several commensal and pathogenic enterobacteria express *bd*-type oxidases, likely stimulated by the milieu present in the human gut. Moreover, they can be exploited in environmental biotechnological applications aimed at increasing bacterial resistance to ammonia.

The *bd*-II enzyme belongs to a hitherto uncharacterized group of *bd*-type terminal oxidases that are increasingly attracting research attention. This work uncovers a novel physiological role for the *E. coli* cytochrome *bd*-II, providing insight into the mechanisms underlying ammonia-induced acceleration of its catalytic activity. These data are also of interest from an evolutionary perspective. Phylogenetic analyses indicate that *bd*-type oxidases originated about 2.4 billion years ago when the Great Oxidation Event led to an increase in O_2_ levels [80,81]. During that period, the atmospheric O_2_ fluctuated at between 0 and 2% for 1.7 billion years, while ammonia levels in the oceans remained elevated, with concentrations significantly higher than those of modern times [82]. Changes in O_2_ and other gases’ availability acted as a strong evolutionary driver, requiring organisms to adapt profoundly to survive. It is highly likely that *bd*-type oxidases were crucial in this evolutionary landscape [21]. As these enzymes enable bacteria to utilize O_2_ despite high ammonia levels, they may have provided a major survival advantage to microbes inhabiting environments where both chemicals co-existed.

## Data Availability

The data presented in this study are contained in the article and the Supplementary Materials.

## Supplementary Materials

The following supporting information can be downloaded at https://www.mdpi.com/article/10.3390/antiox15070859/s1, Figure S1: Effect of ammonia on O_2_ consumption by *E. coli* cytochrome *bd-*II in different environments at pH 7.0.

## Abbreviations

The following abbreviations are used in this manuscript:

*A*_max_	Theoretical maximum percent activity
K_d_*_app_*	Apparent dissociation constant
DTT	Dithiothreitol
Q_1_	2,3-dimethoxy-5-methyl-6-(3-methyl-2-butenyl)-1,4-benzoquinone

## References

[1] Poole R.K., Cook G.M. (2000). Redundancy of aerobic respiratory chains in bacteria? Routes, reasons and regulation. Adv. Microb. Physiol..

[2] Kaila V.R.I., Wikstrom M. (2021). Architecture of bacterial respiratory chains. Nat. Rev. Microbiol..

[3] Patil A.V., Shirsath A.M., Anand A. (2024). Dioxygen reductase heterogeneity is crucial for robust aerobic growth physiology of Escherichia coli. iScience.

[4] Borisov V.B., Verkhovsky M.I. (2015). Oxygen as Acceptor. EcoSal Plus.

[5] Pereira M.M., Santana M., Teixeira M. (2001). A novel scenario for the evolution of haem-copper oxygen reductases. Biochim. Biophys. Acta.

[6] Murali R., Hemp J., Gennis R.B. (2022). Evolution of quinol oxidation within the heme-copper oxidoreductase superfamily. Biochim. Biophys. Acta Bioenerg..

[7] Shimada A., Tsukihara T., Yoshikawa S. (2026). The three-dimensional structure of a proton-pumping pathway, the H-pathway, is evolutionarily conserved in all three families of cytochrome *c* oxidase. Front. Chem..

[8] Murali R., Gennis R.B., Hemp J. (2021). Evolution of the cytochrome *bd* oxygen reductase superfamily and the function of CydAA’in Archaea. ISME J..

[9] Borisov V.B., Siletsky S.A., Paiardini A., Hoogewijs D., Forte E., Giuffre A., Poole R.K. (2021). Bacterial oxidases of the cytochrome *bd* family: Redox enzymes of unique structure, function and utility as drug targets. Antioxid. Redox Signal..

[10] Puustinen A., Finel M., Haltia T., Gennis R.B., Wikstrom M. (1991). Properties of the two terminal oxidases of *Escherichia coli*. Biochemistry.

[11] Borisov V.B., Murali R., Verkhovskaya M.L., Bloch D.A., Han H., Gennis R.B., Verkhovsky M.I. (2011). Aerobic respiratory chain of *Escherichia coli* is not allowed to work in fully uncoupled mode. Proc. Natl. Acad. Sci. USA.

[12] Abramson J., Riistama S., Larsson G., Jasaitis A., Svensson-Ek M., Laakkonen L., Puustinen A., Iwata S., Wikstrom M. (2000). The structure of the ubiquinol oxidase from *Escherichia coli* and its ubiquinone binding site. Nat. Struct. Biol..

[13] Li J., Han L., Vallese F., Ding Z., Choi S.K., Hong S., Luo Y., Liu B., Chan C.K., Tajkhorshid E. (2021). Cryo-EM structures of *Escherichia coli* cytochrome *bo_3_* reveal bound phospholipids and ubiquinone-8 in a dynamic substrate binding site. Proc. Natl. Acad. Sci. USA.

[14] Gao Y., Zhang Y., Hakke S., Mohren R., Sijbers L., Peters P.J., Ravelli R.B.G. (2024). Cryo-EM structure of cytochrome *bo*_3_ quinol oxidase assembled in peptidiscs reveals an “open” conformation for potential ubiquinone-8 release. Biochim. Biophys. Acta Bioenerg..

[15] Guo Y., Karimullina E., Emde T., Otwinowski Z., Borek D., Savchenko A. (2023). Monomer and dimer structures of cytochrome *bo_3_* ubiquinol oxidase from *Escherichia coli*. Protein Sci..

[16] Safarian S., Hahn A., Mills D.J., Radloff M., Eisinger M.L., Nikolaev A., Meier-Credo J., Melin F., Miyoshi H., Gennis R.B. (2019). Active site rearrangement and structural divergence in prokaryotic respiratory oxidases. Science.

[17] Thesseling A., Rasmussen T., Burschel S., Wohlwend D., Kagi J., Muller R., Bottcher B., Friedrich T. (2019). Homologous *bd* oxidases share the same architecture but differ in mechanism. Nat. Commun..

[18] van der Velden T.T., Kayastha K., Pelser F., Brunle S., Jeuken L.J.C. (2026). Visualizing the mechanism of quinol oxidation and inhibition of a *bd*-type oxidase using cryo-EM. Sci. Adv..

[19] Grauel A., Kagi J., Rasmussen T., Makarchuk I., Oppermann S., Moumbock A.F.A., Wohlwend D., Muller R., Melin F., Gunther S. (2021). Structure of *Escherichia coli* cytochrome *bd*-II type oxidase with bound aurachin D. Nat. Commun..

[20] Grund T.N., Radloff M., Wu D., Goojani H.G., Witte L.F., Josting W., Buschmann S., Muller H., Elamri I., Welsch S. (2021). Mechanistic and structural diversity between cytochrome *bd* isoforms of *Escherichia coli*. Proc. Natl. Acad. Sci. USA.

[21] Borisov V.B., Giardina G., Pistoia G., Forte E. (2025). Cytochrome *bd*-type oxidases and environmental stressors in microbial physiology. Adv. Microb. Physiol..

[22] Lu P., Heineke M.H., Koul A., Andries K., Cook G.M., Lill H., van Spanning R., Bald D. (2015). The cytochrome *bd*-type quinol oxidase is important for survival of *Mycobacterium smegmatis* under peroxide and antibiotic-induced stress. Sci. Rep..

[23] Seregina T.A., Lobanov K.V., Shakulov R.S., Mironov A.S. (2022). Inactivation of terminal oxidase bd-I leads to supersensitivity of *E. coli* to quinolone and beta-lactam antibiotics. Mol. Biol..

[24] Engelgeh T., Wamp S., Rothe P., Herrmann J., Fischer M.A., Muller R., Halbedel S. (2025). ClpP2 proteasomes and SpxA1 determine *Listeria monocytogenes* tartrolon B hyper-resistance. PLoS Genet..

[25] Zhuang H.H., Chen Y., Hu Q., Long W.M., Wu X.L., Wang Q., Xu T.T., Qu Q., Liu Y.P., Xiao Y.W. (2023). Efficacy and mortality of ceftazidime/avibactam-based regimens in carbapenem-resistant Gram-negative bacteria infections: A retrospective multicenter observational study. J. Infect. Public Health.

[26] Zhao Y.L., Qu Q., Wang Y.M., Zhang Y.T., Qu J. (2026). A disproportionality analysis of adverse events associated with omadacycline based on the FDA adverse event reporting system database. J. Antimicrob. Chemother..

[27] Roy A., Naik D., Sau S., Agnivesh P.K., Parida K.K., Kalia N.P. (2026). Simultaneous Inhibition of Cytochrome *bd* Oxidase and ATP Synthase for Complete Eradication of *Mycobacterium tuberculosis*. ACS Infect. Dis..

[28] Kovalova T., Janczak M., Gamiz-Hernandez A.P., Lundin D., Sharma S., Vilhjalmsdottir J., Sjostrand D., Kaila V.R.I., Hogbom M., Adelroth P. (2026). The *Mycobacterium smegmatis bd*-II terminal oxidase employs a carboxylate shift mechanism. Proc. Natl. Acad. Sci. USA.

[29] Rahman N.A., Singh S., Wiggins T., Santos M.D., Moraski G.C., Miller M.J., Berney M., Pethe K. (2026). A bactericidal tuberculosis drug regimen driven by inhibition of the terminal oxidases by pretomanid. EMBO Mol. Med..

[30] Saha P., Kumar M., Sharma D.K. (2026). Inhibitors of type II NADH-dehydrogenase, cytochrome bd oxidase, and ATP synthase for anti-tubercular response. RSC Med. Chem..

[31] Henry S.A., Sansom G.N., Tran T.T.P., Boughton R.A., Joiner G., Webster C.M., de Silva H.I.C., Garrett M.D., Serpell C.J., Robinson G.K. (2026). Investigating the role of cytochrome *bd* oxidases in the antibacterial activity of madecassic acid and derivatives thereof. RSC Med. Chem..

[32] Siddeeque R., Etcheverry B., Cattin C., Deviers J., Melin F., Hellwig P., Cailliez F., de la Lande A. (2026). Computational Study of Heme *b*_595_ to Heme *d* Electron Transfer in *E. coli* Cytochrome *bd*-I Oxidase. J. Chem. Inf. Model..

[33] Gupta P., Caldbeck R., Walters R.C., Wells E.C., Hardman B.L., Christie G., Springett R.J., Blaza J.N. (2025). Early activation of bioenergetic metabolism powers bacterial spore germination. Proc. Natl. Acad. Sci. USA.

[34] Krol S., Kovalova T., Janczak M., Kalsum S., Akber M., Hogbom M., Brighenti S., Adelroth P., Brzezinski P. (2025). Mycobacterial respiratory chain enzymes and growth are inhibited by decylubiquinone. Commun. Biol..

[35] Siddeeque R., Heger L., Kagi J., Friedrich T., Melin F., Hellwig P. (2025). Interplay of acidic residues in the proton channel of E. coli cytochrome bd-I oxidase to promote oxygen reduction and NO release. Biochim. Biophys. Acta Bioenerg..

[36] Xiao X., Song G., Lu H., Zheng W., Meng P., Peng W., Yang J., Wang M., Zhu J., Wang J. (2025). Cytochrome bd-II oxidase CyxA promotes the pathogenicity of Klebsiella pneumoniae by resisting oxidative stress. Virulence.

[37] Larabi A.B., Tiffany C.R., Masson H.L.P., Nguyen H., Bejarano E.J., Liou M.J., Radlinski L.C., Demars A.M., Tsolis R.M., Baumler A.J. (2025). Salmonella produces sulfide to compete with Escherichia coli in the gut lumen. Proc. Natl. Acad. Sci. USA.

[38] Wu X., Zhang X., Xia W., Zhang Y., Huang K., Zhao F., Ji C., Wang J., Zhou B., Zhang J.Z.H. (2025). Discovery of novel Cytochrome *bd* oxidase inhibitors against *Mycobacterium tuberculosis*. Eur. J. Med. Chem..

[39] Kumar R., Roy A., Kalia N.P., Sharma D.K. (2025). Inhibition of cytochrome *bd* oxidase in *Mycobacterium tuberculosis* by benzothiazole amides. Bioorg. Med. Chem..

[40] Afarin M., Naeimpoor F. (2025). Diazotrophic growth of free-living *Rhizobium etli*: Community-like metabolic modeling of growing and non-growing nitrogen-fixing cells. PLoS ONE.

[41] Clariano M., Nunes D., Canudo D., Macas D., Castro B.J.L., Jordaan A., Gomes P., Contini A., Perdigao J., Portugal I. (2025). Pyrroloquinolone-Based Compounds as a Novel Antimycobacterial Chemotype. ACS Med. Chem. Lett..

[42] Murillo-Bello A., Ricciardulli A.G., Umar A.R., Gerasimova T., Friedrich T., Javahiraly N., Samori P., Hellwig P. (2025). Graphene Nanodots as Substrates for SEIRAS and SERS Studies on Membrane Proteins. Langmuir.

[43] Buglino J.A., Ozakman Y., Hatch C.E., Benjamin A., Tan D.S., Glickman M.S. (2025). Chalkophore-mediated respiratory oxidase flexibility controls *M. tuberculosis* virulence. eLife.

[44] Mathiyazakan V., Kathalingam S.S., Pok W.N., Sorayah R., Pethe K., Gruber G. (2025). Whole-Cell Study Unveils Critical Mechanistic Elements, Regulatory Elements, and Inhibitor Targets of the Mycobacterium abscessus Cytochrome bd Oxidase. ACS Infect. Dis..

[45] Hellwig P. (2025). The electrochemical properties of the highly diverse terminal oxidases from different organisms. Bioelectrochemistry.

[46] Batista B.B., de Lima V.M., Will W.R., Fang F.C., da Silva Neto J.F. (2025). A cytochrome *bd* repressed by a MarR family regulator confers resistance to metals, nitric oxide, sulfide, and cyanide in *Chromobacterium violaceum*. Appl. Environ. Microbiol..

[47] Inskeep W.P., Jay Z.J., McKay L.J., Dlakic M. (2025). Respiratory processes of early-evolved hyperthermophiles in sulfidic and low-oxygen geothermal microbial communities. Nat. Commun..

[48] Janczak M., Vilhjalmsdottir J., Adelroth P. (2024). Proton transfer in cytochrome bd-I from E. coli involves Asp-105 in CydB. Biochim. Biophys. Acta Bioenerg..

[49] Tu Z., Stevenson D.M., McCaslin D., Amador-Noguez D., Huynh T.N. (2024). The role of *Listeria monocytogenes* PstA in beta-lactam resistance requires the cytochrome *bd* oxidase activity. J. Bacteriol..

[50] Hao H., Nie Z., Wu Y., Liu Z., Luo F., Deng F., Zhao L. (2024). Probiotic Characteristics and Anti-Inflammatory Effects of *Limosilactobacillus fermentum* 664 Isolated from Chinese Fermented Pickles. Antioxidants.

[51] Seitz C., Ahn S.H., Wei H., Kyte M., Cook G.M., Krause K.L., McCammon J.A. (2024). Targeting Tuberculosis: Novel Scaffolds for Inhibiting Cytochrome bd Oxidase. J. Chem. Inf. Model..

[52] Saha P., Das S., Indurthi H.K., Kumar R., Roy A., Kalia N.P., Sharma D.K. (2024). Cytochrome *bd* oxidase: An emerging anti-tubercular drug target. RSC Med. Chem..

[53] McKay L.S., Camilli A., Cotter P.A. (2026). The role of cytochrome oxidases in bacterial virulence. Infect. Immun..

[54] Li J., Gao P., Kao R.Y., Li H., Sun H. (2026). Bismuth drug as an antibiotic adjuvant to inhibit biofilm formation via a dual mechanism. RSC Med. Chem..

[55] Wrong O. (1978). Nitrogen metabolism in the gut. Am. J. Clin. Nutr..

[56] Fried D.E., Watson R.E., Robson S.C., Gulbransen B.D. (2017). Ammonia modifies enteric neuromuscular transmission through glial gamma-aminobutyric acid signaling. Am. J. Physiol. Gastrointest. Liver Physiol..

[57] Eklou-Lawson M., Bernard F., Neveux N., Chaumontet C., Bos C., Davila-Gay A.M., Tome D., Cynober L., Blachier F. (2009). Colonic luminal ammonia and portal blood L-glutamine and L-arginine concentrations: A possible link between colon mucosa and liver ureagenesis. Amino Acids.

[58] Forte E., Siletsky S.A., Borisov V.B. (2021). In *Escherichia coli* ammonia inhibits cytochrome *bo*_3_ but activates cytochrome *bd*-I. Antioxidants.

[59] Forte E., Borisov V.B., Falabella M., Colaco H.G., Tinajero-Trejo M., Poole R.K., Vicente J.B., Sarti P., Giuffre A. (2016). The terminal oxidase cytochrome *bd* promotes sulfide-resistant bacterial respiration and growth. Sci. Rep..

[60] Borisov V., Arutyunyan A.M., Osborne J.P., Gennis R.B., Konstantinov A.A. (1999). Magnetic circular dichroism used to examine the interaction of *Escherichia coli* cytochrome *bd* with ligands. Biochemistry.

[61] Lorence R.M., Miller M.J., Borochov A., Faiman-Weinberg R., Gennis R.B. (1984). Effects of pH and detergent on the kinetic and electrochemical properties of the purified cytochrome *d* terminal oxidase complex of *Escherichia coli*. Biochim. Biophys. Acta.

[62] Kelley R.W., Reed J.R., Backes W.L. (2005). Effects of ionic strength on the functional interactions between CYP2B4 and CYP1A2. Biochemistry.

[63] Wang R. (2014). Gasotransmitters: Growing pains and joys. Trends Biochem. Sci..

[64] Yuan Y., Zhang W., Li X., Ge H., Zhu H. (2026). Ammonia signaling network: The intersection of tumor metabolism, epigenetics, and metastasis. Front. Endocrinol..

[65] Chen G., Wang C., Huang S., Yang S., Su Q., Wang Y., Dai W. (2025). Novel roles of ammonia in physiology and cancer. J. Mol. Cell. Biol..

[66] Karababa A., Gorg B., Schliess F., Haussinger D. (2014). *O*-GlcNAcylation as a novel ammonia-induced posttranslational protein modification in cultured rat astrocytes. Metab. Brain Dis..

[67] Komatsu A., Iida I., Nasu Y., Ito G., Harada F., Kishikawa S., Moss S.J., Maeda T., Terunuma M. (2022). Ammonia induces amyloidogenesis in astrocytes by promoting amyloid precursor protein translocation into the endoplasmic reticulum. J. Biol. Chem..

[68] Dabrowska K., Skowronska K., Popek M., Albrecht J., Zielinska M. (2021). The Role of Nrf2 Transcription Factor and Sp1-Nrf2 Protein Complex in Glutamine Transporter SN1 Regulation in Mouse Cortical Astrocytes Exposed to Ammonia. Int. J. Mol. Sci..

[69] Bernier S.P., Letoffe S., Delepierre M., Ghigo J.M. (2011). Biogenic ammonia modifies antibiotic resistance at a distance in physically separated bacteria. Mol. Microbiol..

[70] Dasarathy S., Mookerjee R.P., Rackayova V., Rangroo Thrane V., Vairappan B., Ott P., Rose C.F. (2017). Ammonia toxicity: From head to toe?. Metab. Brain Dis..

[71] Oleskin A.V., Shenderov B.A. (2016). Neuromodulatory effects and targets of the SCFAs and gasotransmitters produced by the human symbiotic microbiota. Microb. Ecol. Health Dis..

[72] Vertzoni M., Augustijns P., Grimm M., Koziolek M., Lemmens G., Parrott N., Pentafragka C., Reppas C., Rubbens J., Van Den Alphabeele J. (2019). Impact of regional differences along the gastrointestinal tract of healthy adults on oral drug absorption: An UNGAP review. Eur. J. Pharm. Sci..

[73] Forte E., Borisov V.B., Siletsky S.A., Petrosino M., Giuffre A. (2019). In the respiratory chain of *Escherichia coli* cytochromes *bd*-I and *bd*-II are more sensitive to carbon monoxide inhibition than cytochrome *bo*_3_. Biochim. Biophys. Acta Bioenerg..

[74] Nastasi M.R., Borisov V.B., Forte E. (2024). Membrane-bound redox enzyme cytochrome *bd*-I promotes carbon monoxide-resistant *Escherichia coli* growth and respiration. Int. J. Mol. Sci..

[75] Belevich I., Borisov V.B., Verkhovsky M.I. (2007). Discovery of the true peroxy intermediate in the catalytic cycle of terminal oxidases by real-time measurement. J. Biol. Chem..

[76] Paulus A., Rossius S.G., Dijk M., de Vries S. (2012). Oxoferryl-porphyrin radical catalytic intermediate in cytochrome *bd* oxidases protects cells from formation of reactive oxygen species. J. Biol. Chem..

[77] Borisov V.B., Forte E., Sarti P., Giuffre A. (2011). Catalytic intermediates of cytochrome *bd* terminal oxidase at steady-state: Ferryl and oxy-ferrous species dominate. Biochim. Biophys. Acta.

[78] Vlasova I.I. (2018). Peroxidase activity of human hemoproteins: Keeping the fire under control. Molecules.

[79] Belevich I., Borisov V.B., Bloch D.A., Konstantinov A.A., Verkhovsky M.I. (2007). Cytochrome *bd* from *Azotobacter vinelandii*: Evidence for high-affinity oxygen binding. Biochemistry.

[80] Trost K., Gennis R.B., Allen J.F., Mills D.B., Martin W.F. (2026). Oxygen reductase origin followed the great oxidation event and terminated the Lomagundi excursion. Biochim. Biophys. Acta Bioenerg..

[81] Degli Esposti M., Mentel M., Martin W., Sousa F.L. (2019). Oxygen Reductases in Alphaproteobacterial Genomes: Physiological Evolution from Low to High Oxygen Environments. Front. Microbiol..

[82] Zerkle A.L., Poulton S.W., Newton R.J., Mettam C., Claire M.W., Bekker A., Junium C.K. (2017). Onset of the aerobic nitrogen cycle during the Great Oxidation Event. Nature.

