# Supplementary material for: Effect of Ammonia on Escherichia coli Growth and Aerobic Respiration: The Role of Cytochrome bd-II
Source: Antioxidants (Basel). 2026 Jul 9;15(7):859. doi: 10.3390/antiox15070859 (PMC13405924; doi:10.3390/antiox15070859)
Supplement: Supplementary file 1 [file antioxidants-15-00859-s001.zip › antioxidants-4405343-supplementary.pdf]

Supplementary Materials for

Article

Effect of Ammonia on *Escherichia coli* Growth and Aerobic Respiration: The Role of Cytochrome *bd*-II

Francesca Giordano [1], Martina Roberta Nastasi [1], Vitaliy B. Borisov [2,3,*] and Elena Forte [1,*]

[1] Department of Biochemical Sciences, Sapienza University of Rome, I-00185 Rome, Italy
[2] Belozersky Institute of Physico-Chemical Biology, Lomonosov Moscow State University, Leninskie Gory, 119991 Moscow, Russia
[3] Faculty of Bioengineering and Bioinformatics, Lomonosov Moscow State University, Leninskie Gory, 119991 Moscow, Russia
* Correspondence: bor@belozersky.msu.ru (V.B.B.); elena.forte@uniroma1.it (E.F.)

Supplementary Figure

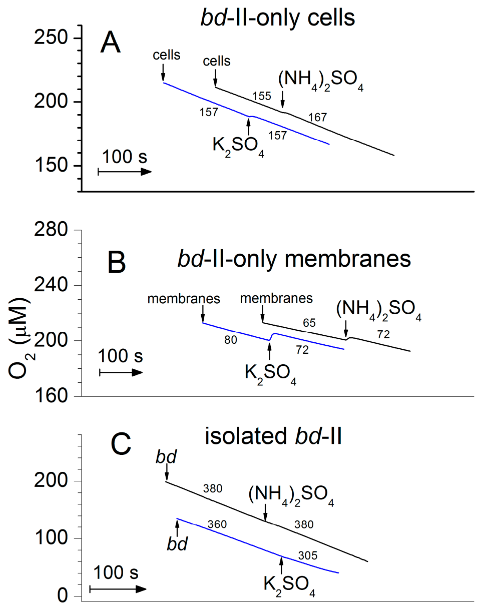

Supplementary Figure S1. Effect of ammonia on O_2 consumption by *E. coli* cytochrome *bd*-II in different environments at pH 7.0. (A) *bd*-II-only cells (1 mL, OD_{600} = 1.7). (B) Membranes from *bd*-II-only cells (80 μg protein·mL^{-1}). (C) isolated cytochrome *bd*-II (2.5 nM). Representative O_2 consumption traces are shown. O_2 consumption rates (nM O_2·s^{-1}), measured before and after the addition of either ammonia or K_2SO_4 (ionic strength control) are indicated next to each trace. Data are representative of three independent experiments (*n* = 3). NH_3 concentrations of 0.3 mM (A), 0.56 mM (B), and 0.14 mM (C) were generated by the addition of 27, 50, and 12.5 mM (C) $(NH_4)_2SO_4$, respectively. Corresponding K_2SO_4 concentrations used as controls were 27 mM (A), 50 mM (B), and 12.5 mM (C).
